# Wayfinding as a Social Activity

**DOI:** 10.3389/fpsyg.2019.00142

**Published:** 2019-02-04

**Authors:** Ruth C. Dalton, Christoph Hölscher, Daniel R. Montello

**Affiliations:** ^1^Department of Architecture and Built Environment, University of Northumbria at Newcastle, Newcastle upon Tyne, United Kingdom; ^2^Department of Humanities, Social and Political Sciences, ETH Zurich, Zurich, Switzerland; ^3^Department of Geography, University of California, Santa Barbara, Santa Barbara, CA, United States

**Keywords:** social wayfinding, navigation, group decision making, spatial cognition, wayfinding taxonomy

## Abstract

We discuss the important, but greatly under-researched, topic of the social aspects of human wayfinding during navigation. Wayfinding represents the planning and decision-making component of navigation and is arguably among the most common, real-world domains of both individual and group-level decision making. We highlight the myriad ways that wayfinding by people is not a solitary psychological process but is influenced by the actions of other people, even by their mere presence. We also present a novel and comprehensive framework for classifying wayfinding in complex environments that incorporates the influence of other people. This classification builds upon the premises of previous wayfinding taxonomies and is further structured into four parts based upon (1) the nature of the interaction between the actors and (2) the time frame in which the interaction takes place. We highlight gaps in our current understanding of social wayfinding and outline future research opportunities.

## Introduction

The study of human wayfinding during navigation is central to research on human spatial cognition. Navigation is a routine, everyday activity and draws upon numerous cognitive functions, including perception, memory (declarative and non-declarative), imagination, language, reasoning, and decision-making. Emotion is involved too, as when anxiety impairs spatial decision-making during emergency egress or when landmarks are more readily recalled when they have a positive emotional association (Gartner, 2011, 2012; Palmiero and Piccardi, 2017; Ruotolo et al., 2018). Human and animal navigation has been investigated by different research communities (cognitive, developmental and environmental psychology; geography; anthropology; linguistics; architecture; animal behavior; neuroscience; robotics and artificial intelligence [AI]), each with different emphases and methods. We distinguish “locomotion,” the movement of one’s body coordinated to the proximal environment, from “wayfinding,” the planning required for efficient and goal-directed navigation (Montello, 2005; Montello and Sas, 2006)^1^. Locomotion depends on sensori-motor systems interacting with an immediate surrounding, while wayfinding invokes higher-level, cognitive systems to maintain orientation relative to the distal environment. In other words, wayfinding involves figuring out where you are, where you want to go and how to get there, particularly when your goal cannot be directly sensed at that moment. In this paper, we focus on the wayfinding component of navigation.

A considerable range of environmental features influences the decision-making of navigators in real-world environments. Research on spatial cognition from environmental psychology, behavioral geography, and related disciplines has mostly concentrated on human-made entities in the environment, such as buildings or urban street networks, and information displays, including signage and maps. Wayfinding research in cognitive psychology and neuroscience has concentrated on the internal processes of how individual wayfinders represent and memorize landmarks and spatial arrangements. But a common feature shared by nearly all wayfinding studies is that they do not take the co-presence, and potential influence, of other human beings into account. The present paper aims to define and explicate the contributions that social “others” may have in wayfinding processes. We conclude that these contributions are extensive and intricate in nature, and that their oversight thus far has distorted our understanding of wayfinding processes. A host of fascinating issues are raised by our analysis, pointing to ample potential for further research on the social aspects of wayfinding.

Wayfinding can be described fundamentally as a decision-making process (Passini, 1981). It is a prototypical real-world example of complex cognition (Sternberg and Ben-Zeev, 2001) as it builds upon people’s perception of the environment, memory of past experiences, spatial learning processes, motor processes, and inferential as well as emotional appraisal of navigation options. Social aspects, i.e., the co-presence of, or interaction with, other people, can influence the heuristics, strategies, and expectations of the wayfinder (e.g., Zacharias, 2001).

In the broadest sense, any situation where the presence and/or activities of others, now or in the past, has an observable impact on wayfinding behavior and cognition can be called “social wayfinding.” This suggests the great breadth—even ubiquity—of the influence of the *social* in wayfinding. Even when other people are not directly present during navigation or have not provided any information directly relevant to a traveler’s route choice, it is clear that other individuals, social groups, institutions, and cultural practices always exert *some influence* on the psychology of wayfinding. Tackling such an exceedingly broad definition of social wayfinding is beyond the scope of this paper, hence the following section outlines those aspects of social wayfinding excluded from this paper.

### Aspects of Social Wayfinding Not Covered by This Paper

Especially when one considers social wayfinding in the broad sense we have just outlined, a comprehensive overview would encompass far too much for a single manuscript, perhaps even too much for a single book. One way we delimit our analysis here is to focus specifically on situations where other people directly or indirectly determine the specific routes a navigator chooses while traveling. This still leaves us with a very broad and diverse topic of interest. In many cases, it includes situations wherein other people are present where and when the travel is occurring. But we also include situations wherein the other people are not present at the time and place of travel, but their activities in the past influence the specific wayfinding choices of a navigator. We discuss both of these situations further below.

The design of navigation technologies and artifacts (maps, compasses, sextants, GPS) always reflects, in part, the past decisions and influences of other people (Hutchins, 1995; MacEachren, 1995). Both built spaces and signage are typically designed and positioned by other people, sometimes following regulatory guidelines or widespread cultural practices (Weisman, 1981; Hillier and Hanson, 1984; Passini, 1992). The designers/designs of such navigational artifacts are *not* within the scope of this paper.

Another group that falls into this *most broad sense* might be termed “others as sources of wisdom”; some navigation strategies we employ have been learned from instruction by others (in person or from texts). Even in the least social case of solo travel, without maps, in wilderness environments, people make wayfinding decisions based, in part, on advice they have acquired from others (“*go to a high point*”). In this paper, we do not attempt to cover the full spectrum of the role of the social in wayfinding since it would contain too much material for a single manuscript.

**FIGURE 1:**
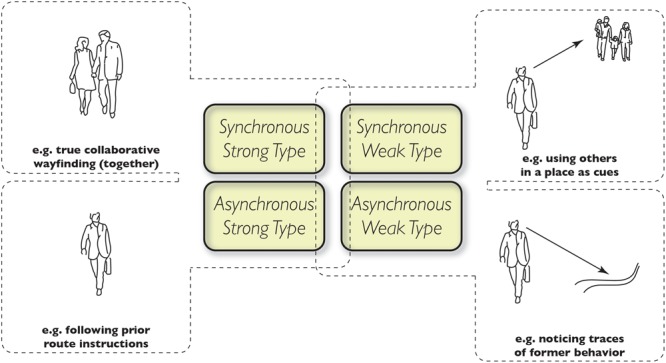
The four types of social wayfinding: synchronous and strong **(upper left)**; asynchronous and strong **(lower left)**; synchronous and weak **(upper right)**; asynchronous and weak **(lower right)**.

Finally, some aspects of social wayfinding, such as the offering of route directions, most certainly fall into our core definition of social wayfinding (these would fall into the bottom-left quadrant of our taxonomy, as put forward in the next section, and illustrated in Figure 1), however, since these are covered reasonably thoroughly elsewhere (e.g., Allen, 1997; Denis et al., 1999; Denis, 2018) we are electing not to include an overview of the route directions literature within this paper. We are, instead, focusing this paper on research that is not yet comprehensively described in the spatial cognition literature; in the next section we will outline the scope of social wayfinding as included in this paper.

### Scope of Paper

Our analysis of social wayfinding focuses specifically on situations where other people directly or indirectly determine the specific routes a navigator chooses while traveling. This still leaves a very broad and diverse topic of interest. Primarily this includes situations where others are co-present during travel. But we also include situations wherein others are not present at the time and place of travel, but their activities in the past influence the specific wayfinding choices of a navigator. To understand different modes of social wayfinding, we need to scrutinize the degree, nature, and temporal-frame of the influence exerted on the wayfinder.

Perhaps the clearest case of social wayfinding is given when two or more people actively communicate while navigating to jointly identify and plan a route from A to B, and they both (or all) wish to jointly reach that destination B. At this most prototypical level, the activity can be defined as *traveling in a group* whilst interacting to determine route choices. However, this basic activity becomes less social when one individual inadvertently or intentionally dominates the group-interaction or when one individual is explicitly assigned the role of leader (e.g., the tour guide for an excursion). In these cases, the wayfinding almost reduces to a case of individualistic cognition, and the role of the other travelers is largely reduced to following the orders/instructions/route of the leader.

At the other end of the spectrum, social wayfinding can result from an indirect influence: a wayfinder may use the co-presence of others as a kind of environmental cue that provides hints about the suitability of available route choices toward the destination. For example, a wayfinder may choose a route option depending on how busy it is, such as a route through a train station or public street. If the wayfinder believes that many of the other travelers are likely to share the same destination (e.g., from the train station to the football stadium or from the airplane to baggage claim), he or she may simply “go with the flow.” If, by contrast, the wayfinder assumes that the others are less likely to share the destination, he or she may choose to ignore that cue or even deliberately choose an alternative path to avoid being held up by a slow crowd.

### Existing Research on Social Wayfinding

The few studies we review below notwithstanding, we believe the contribution of other people to wayfinding decision-making by humans has been distinctly under-researched. A very large number of wayfinding studies with humans have been conducted, both experimental and non-experimental, in both laboratory and field settings (to cite just a few: Bronzaft et al., 1976; Kaplan, 1976; Bovy and Stern, 1990; Peponis et al., 1990; Cornell et al., 1992; Golledge, 1999; Maguire et al., 2000; Carpman and Grant, 2002; McNamara et al., 2008; Hill, 2013). In spite of its extensive and rich history, almost none of this research has studied wayfinding as a social problem but almost always as a problem for single individuals. This is certainly no less true of the research we ourselves have done on wayfinding (e.g., Montello and Pick, 1993; Conroy Dalton, 2003; Hölscher et al., 2006). Even in the fields of agent-based modeling, simulated pedestrian agents tend to be solitary and do not include higher-order groups, or if they do, the groups are merely co-located agents, moving as a single “unit” rather than interacting meaningfully as a group (McDermott and Davis, 1984; Yeap and Jefferies, 1999; Kuipers, 2000; Raubal, 2001).

Why has the contribution of other people to wayfinding decisions been so under-researched? One reason is undoubtedly the intellectual penchant in experimental psychology for seeking understanding at the individualistic level of analysis (Brennan, 2003). Another must be the extra complexity implied by analyzing a decision-making process involving two or more individuals—with its potential for emergent interactions between the individuals—instead of analyzing it solely at the individual level. Another, more contemporary, reason for the neglect of the social in wayfinding may be the increasing use of virtual-reality (VR) technologies as experimental tools and settings for wayfinding research (e.g., Péruch et al., 1997; Ruddle et al., 1997; Steck and Mallot, 2000; Meilinger et al., 2008). These experimental environments are typically devoid of other “people,” although they do not need to be.

An important exception to our claim that social aspects of wayfinding have been relatively neglected is the substantial body of research on verbal wayfinding instructions (“route directions”) (Klein, 1983; Allen, 1997; Denis et al., 1999; Lovelace et al., 1999; Hölscher et al., 2011; Denis, 2018). We omit a detailed review of literature on giving and interpreting verbal route directions here, in part to keep our manuscript tractable but also because it is covered fairly thoroughly elsewhere, as we have just cited; research on route directions covers only a small, albeit important, portion of the issues surrounding social wayfinding. In fact, most research on wayfinding instructions only implicitly involves the social aspects of wayfinding, the involvement of multiple people (or navigation software created by people) notwithstanding. Most of this research instead focuses on the informational content provided in the instructions (i.e., how many words, landmarks, metric statements, etc., the instructions contain). Wunderlich and Reinelt (1982) is a notable exception, concentrating on the nature of the dialog between two people in a conversational exchange about routes. So we certainly do include wayfinding instructions as a case of social wayfinding, but we do not discuss it much here as it is thoroughly discussed in other literature (e.g., the citations above).

Besides the work on wayfinding instructions, we find very little research on the social aspects of wayfinding decision-making. A few studies have come out recently on social interaction during collective navigation. Reilly et al. (2009) looked at the use of mobile devices (smart phones) during navigation, although the emphasis in this study was on how the device is shared rather than on how collaborative wayfinding decisions are made. A few other studies have focused on social wayfinding that is more directly collaborative. Forlizzi et al. (2010) observed the interaction between members of dyads (couples) traveling while using in-vehicle navigation systems, offering suggestions for system design following from their observations of wayfinding interactions. Haddington (2012, 2013) conducted an interesting study on gestures and conversational interchanges about navigation among people riding in a car. Similarly, He et al. (2015) observed pairs of individuals walking routes in an unfamiliar urban setting. The two members simultaneously walked two different routes while they gave each other instructions over cellphones about how to follow the route being traversed by the other member. These studies observe ongoing social interaction among actors who are navigating together, focusing on interacting dyads in particular contexts. We believe this work is a promising start but, as we discuss below, we also believe social wayfinding goes well beyond groups of dyads and also well beyond synchronous interaction between members of groups who are concurrently traveling.

An interesting study that does go beyond dyads and considers the possible social interactions of multiple wayfinding people was undertaken by Haghani and Sarvi (2017), who were looking at the role of social behaviors during simulated emergency evacuations. They found that “*navigational escape strategies of humans are… significantly influenced by both physical factors of the escape environment… and the social interactions*.” (p. 53). However, this paper solely focused on wayfinding behavior in an emergency scenario and may not, therefore, be predictive of behavior in everyday wayfinding tasks.

One of the most important and developed bodies of work on wayfinding that explicitly incorporates the social is the work of Hutchins on “cognition in the wild” (Hutchins, 1995). Above, we cited his ideas about navigation technologies and artifacts as providing a sociocultural context for navigation, but he goes much further in conceptualizing the social in wayfinding. In fact, he had the larger aim in his work of socially contextualizing human cognitive research more broadly than just navigation, but he chose navigation as his main test case (which may be significant in and of itself). Hutchins proposes that human reasoning and problem-solving is fundamentally a social and cultural process, embedded in group communication and symbolic artifacts created by past cultural-group members. His main test case was technical sea-navigation on a ship by members of the U.S. Navy. Navigational decisions arise from a series of communications back and forth along formally established social links between sailors holding specific posts, using particular observations and technologies to contribute information to larger navigational decisions. When functioning well, the components work together to improve the validity, reliability, and resolution of decisions about ship localization, heading, landmark identification, distances, and directions. Hutchins observed that the cognition of groups may be quite different from the cognition of the individuals involved in the group task, and not just the sum of the individual contributions. His work is an exceptional example of research on the social in wayfinding but as will become evident below, it only scratches the surface of this topic.

Finally, there is extensive research on group/social interaction and collaborative decision-making in contexts other than navigation (e.g., summaries in Swap, 1984; Davis, 1992; Ellis and Fisher, 1994). Much of this is in the context of collaborative computing environments, such as research on computer-supported cooperative work (CSCW) and social computing (Munro et al., 1999; Höök et al., 2003; Tougne et al., 2008)^2^. However, the results of broader research on group/team interaction and decision-making have not yet been applied to wayfinding during navigation.

## The Four Types of Social Wayfinding

In this section, we present a novel and comprehensive classification for social wayfinding. Recognizing that all wayfinding decisions can, to a greater or lesser extent, be recognized as incorporating some influence or aspect of the social, we restate our interest here only in situations where other people directly or indirectly influence the specific routes a navigator chooses while traveling. We propose that social wayfinding in this sense can be broadly divided into two types, termed “Strong” and “Weak” social wayfinding. One might also refer to Strong social wayfinding as *collaborative wayfinding* and Weak social wayfinding as *people-as-cues*. Table 1 lists the primary characteristics of these two types of social wayfinding.

**Table 1:** Typical characteristics of the two primary types of social wayfinding: strong type and weak type.

Strong Type (Collaborative)	Weak Type (People-as-Cues)
Direct	Indirect
Reciprocal	Unidirectional
Proximal	Distal
Verbal/gestural	Non-verbal
Acquaintance/s	Stranger/s
Rarely many	Often many

The primary distinction between Strong and Weak social wayfinding is the degree of intentionality characterizing the communication (information exchange) of the two types. Strong social wayfinding involves intentional communication about wayfinding between actors (often co-navigators), wherein one or more individuals intend to communicate information about location or route choice to one or more other individuals. Weak social wayfinding involves unintentional communication about wayfinding between actors (often not co-navigators); for this type, one or more individuals unintentionally communicate information about location or route choice to one or more other individuals. The person sending wayfinding cues as part of Weak social wayfinding is generally unaware of providing this information, of course, but even the recipient may not always be aware they are picking up and following cues from others. Both senders and recipients engaging in Strong social wayfinding are aware of exchanging information^3^.

Strong social wayfinding generally involves direct communication between senders and recipients, most often between individuals in close proximity (although one can use pointing gestures to communicate, at a distance, about directions). Weak social wayfinding can also occur over short distances but very often occurs distally, over great distances (but still within the range of sensory access). At least in the synchronous case (below), the communication occurring as part of Strong social wayfinding frequently involves true *interaction* in that is it is reciprocal—both the sender and the receiver of wayfinding information may contribute at different moments to the communicative exchange (as when the recipient tells the sender how familiar he or she is with a particular place). In contrast, communication during Weak social wayfinding is non-interactional and unidirectional from the sender to the recipient.

The communication that takes place during Strong social wayfinding involves gestures such as pointing and graphics such as sketch maps but very often includes verbal communication, and often between acquaintances rather than strangers. During Weak social wayfinding, in contrast, communication can be gestural or verbal (as when you overhear someone talking about the route he or she will take) but is most often based on seeing where people are or were traveling (hearing can play this role in Weak Synchronous social wayfinding—see below). The contact that occurs as part of Weak social wayfinding is very often between complete strangers. Finally, Strong and Weak social wayfinding can occur both among multiple senders and recipients, not just two individuals, but multiple actors are much more common in the case of Weak social wayfinding. In fact, the locations and headings of sizeable crowds of people in the environment are frequently particularly informative for the Weak type. As we have suggested, these characteristics are not “hard and fast” rules, but they do generally distinguish the two types of social wayfinding in most cases.

We can further expand this classification of the two types of social wayfinding according to the time frames in which they occur. We distinguish two time modes: Synchronous and Asynchronous social wayfinding. The Synchronous mode occurs when the communicative acts of sending and receiving wayfinding information are roughly co-present in time and space. The persons involved during the act are perceptually accessible to each other (or at least the sender is accessible to the receiver), either because of literal co-presence in space or because of telecommunication technologies like telephones. All other social wayfinding communications may be termed Asynchronous; the sender and receiver are not co-present during the information exchange. Table 1 can therefore be expanded into a two-by-two structure, shown in Figure 1. In the following sections, we describe each of the four quadrants of Figure 1 in detail.

### Strong Synchronous Social Wayfinding

Strong Synchronous social wayfinding occurs when the influence of others takes place during navigation. This is Strong Synchronous social wayfinding when, instead of a single person making wayfinding decisions by herself or himself, the person actively makes wayfinding decisions with intentional input from others (please refer to Figure 4, under the category of “navigational assistant”). The others may accompany the navigator, in which case they collectively find their way together. Alternatively the others may provide wayfinding instructions, typically in spoken form, but also by gestural pointing to the surrounding area or on a map. Automatically generated route instructions, consulted during travel, would also count as Strong Synchronous social wayfinding, inasmuch as the system was designed and programmed by another human being. In all these scenarios, the contribution of others is direct, intentional, proximal, and typically verbal and/or gestural. In both scenarios, the others are almost always few in number—one or a small group, not crowds. When the others accompany the navigator, they usually know the person in advance; people who only provide instructions are usually strangers to begin, but the exchange of information is a form of social introduction between people.

**FIGURE 2:**
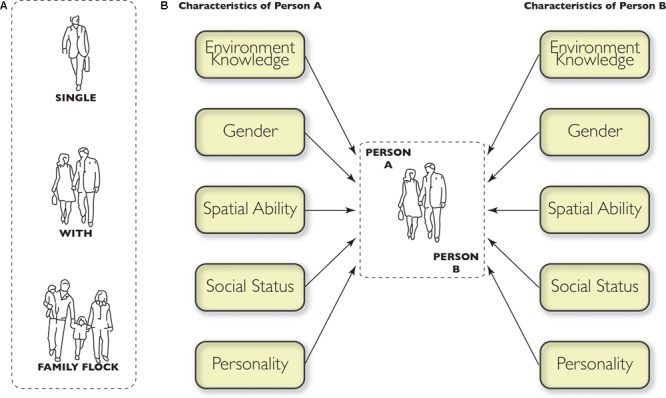
**(A)** Goffman’s classification of pedestrian groups (1971). **(B)** Characteristics of individuals in a group, illustrated as a dyad, (that influence wayfinding interactions). It should be noted that for higher-order groups (three or more), each member of the group will carry such characteristics and possible interactions between group members will be multiplied accordingly. Emergent leadership will thus be important at the group level in order to synchronize wayfinding behaviors.

The sociologist Goffman (1971) undertook seminal theoretical work on the composition and terminology of small social groups (see Figure 2A). According to Goffman, “*a ‘single’ is a party of one, a person who has come alone, a person ‘by himself [herself]’, even though there may be other individuals near him [her] and he [she] has cause for talking to them*.” In contrast, “*a ‘with’ is a party of more than one whose members are perceived to be ‘together’. They maintain some kind of ecological proximity, ensuring the closeness that ordinarily permits easy conversation and the exclusion of non-members who otherwise might intercept talk*.” And, finally, Goffman describes the “family flock” as, “*an example of a ‘with’. A family ‘with’ in a public place will sometimes walk in partial file, or spaced abreast more than a foot apart, making talk a little difficult*.” (Goffman, 1971, pp. 19–20). The accompaniment form of Strong Synchronous social wayfinding is firmly focussed on the wayfinding processes of “withs.”

Helbing and colleagues (Helbing et al., 2000, 2001; for an overview see Moussaid et al., 2009) undertook research on the relative frequency of different sizes of groups of “withs” and found that dyads were the most frequently occurring. Higher-order “withs” decreased in frequency corresponding to the number of people present: Three-person “withs” are less common than dyads but more frequent than four-person groups, etc. Terminology and composition aside, neither Goffman’s nor Helbing’s work are concerned with the types of interaction and communication that take place within groups making wayfinding decisions, but we believe it would be productive to explore their application to this domain.

It is clear that not all groups are equal; we begin with the simplest group, that of a dyad. Some of the studies we cited above have addressed Strong Synchronous social wayfinding by dyads. Reilly et al. (2009) investigated how dyads coordinated the use of a digital map on a mobile phone. They identified the emergence of both leader/follower styles within the dyads as well as collaborative styles wherein dyad members interacted on equal footing and with equal levels of engagement. Of course, there is often a disparity of spatial knowledge within a dyad. One person may be more familiar^4^ with the environment than the other, in which case, you would typically expect the person with greater familiarity to assume more of a leadership role in the collaborative wayfinding [as familiarity is suggested to increase accuracy of direction decisions (Gartner, 2011, 2012)], and the person with less familiarity to tacitly permit himself or herself to be guided, since they would be more likely to make wayfinding errors (Chebat et al., 2005). If both members of the dyad are equally unfamiliar with the environment, then you would expect to find greater mutual decision-making, resulting in more discussion or debate about route choices.

We can also expect that dyads will often be composed of members with different navigational abilities—with a different “sense-of-direction” (Hegarty et al., 2002) or with different “cognitive styles” (Aggarwal and Wooley, 2013). In their study of interacting dyads cited above, He et al. (2015) looked at navigators describing routes to each other over a cellphone as a function of whether both members reported having a good sense-of-direction, both reported having a poor sense-of-direction, or the members reported having a different sense-of-direction (the researchers created the dyads in this way). The researchers found that pairs differing greatly in sense-of-direction found the task more difficult and time consuming, and they observed differences in the types of landmarks mentioned by members providing directions as a function of their sense-of-direction. We speculate that when a person with good sense-of-direction is paired with a partner who has poor sense-of-direction, the better navigator would often assume a more dominant role in the decision-making processes, and the worse navigator a more subservient role. Ultimately, however, belief is more important than reality with variables like familiarity and ability; to assume leadership or subservience, it only matters that one *believes* one is more or less familiar or better or worse at wayfinding (belief and reality do often match with these particular variables). Bonner and Bolinger’s (2013) paper on “Separating the confident from the correct” is a clear example of the differences that can occur between being the most knowledgeable person in the group versus *thinking* one is the most knowledgeable. If these findings are extrapolated to wayfinding situations, it becomes clear how one less knowledgeable or less able wayfinder might lead a whole group astray.

We further expect that other variables characterizing the two individuals in a dyad will moderate the expression of familiarity and sense of direction, such as the gender of the two people, their relative social status, and aspects of their personalities such as extroversion or dominance (summarized in Figure 2B). For instance, if one person is of lower status than the other (a child accompanying a parent, or an employee accompanying a boss), the person of lower status might be loath to question the wayfinding decisions of the higher-status wayfinding partner. Presumably this would happen even if the low-status person believes he or she has superior environmental knowledge or wayfinding abilities. As another example, a more domineering person might naturally assume leadership even though he or she may not be more familiar or able within the dyad. In fact, in their study of dyads interacting while using in-vehicle navigation systems, Forlizzi et al. (2010) observed that the nature of collaborative interactions varied among dyads and was influenced by the individual’s social role, and his or her role as driver or “navigator.” The authors noted that none of these interesting phenomena could be identified by the traditional research paradigm that conceptualizes navigation as a solitary act.

We further expect characteristics of group members to operate differently as a function of the cultural context of the navigation. For example, gender typing is not equivalent in all cultures. Although the notion that males have a better of sense-of-direction^5^ or should take the role of leader in contexts of wayfinding is fairly common across cultures, it is certainly not equally pronounced in all.

The examples thus far consider the simplest form of Goffman’s “with,” namely the dyad. But we certainly believe it is interesting and important to consider groups larger than dyads. Once we get into higher-order “withs,” where each constituent member of the group can be characterized by their gender, environmental familiarity, spatial ability, social status, personality, and so on, it is clear that wayfinding decision-making processes become increasingly complex. Given the complex patterns of interactions between the different variables, traditional empirical methods of observation and measurement would likely be inadequate by themselves to understand emergent effects in larger groups. Detailed computational models would help here, such as spatially and socially intelligent agent-based models.

It is not surprising that intergroup communication during wayfinding occasionally goes awry. Many people have experienced the situation of navigating as part of a pair, whereby each person has implicitly assumed that the other knows where they are going. Each person accordingly but mistakenly assumes a subservient role, only for both parties to eventually realize that neither of the pair are effectively leading, but both are attempting to follow. This situation also highlights the fact that quite often the disparate roles within a wayfinding group are adopted without prior negotiation (i.e., they are assumed or inferred), adding even more complexity to understanding these phenomena.

Individual wayfinders may rely on internal mental capacities only, such as their knowledge and spatial reasoning. By contrast, wayfinders in groups may also have to take into account the preferences of other group members. The degree to which a person adopts the perspective of others and takes into account the effects of their own behavior on the options of other people is referred to as “social mindfulness.” According Van Doesum et al. (2013), this social mindfulness entails affective perspective taking as well as empathic concern. The degree to which such social mindfulness could have an impact on wayfinding decision-making is limited by the willingness to coordinate with another person. Of course, mindfulness for others’ thoughts and feelings, and the social negotiations that attend these concerns, can certainly decrease the efficiency of group decision-making compared to solo decision-making. Interestingly, such concern can even make group decision-making less *effective*, because of the distraction of considering others, conflicts between alternative views that are resolved sub-optimally, and so on. Clearly, we should not expect that two heads are always better than one when people co-wayfind (Hill, 1982; Burte, 2004). It should be clear from the discussion in this section that in order to fully investigate and come to understand collaborative group wayfinding, we will need to draw upon the expertise of a wide-range of academic disciplines, with the diversity of their concepts and methods.

### Strong Asynchronous Social Wayfinding

Strong social wayfinding can also be performed asynchronously, wherein communication about wayfinding decisions occur at a time prior to the actual travel (typically in another place, as well). An example of this would be instructions given to the wayfinder by another person or navigation system, instructions that are written, spoken, or gestured, with or without the aid of commercial maps (i.e., personalized or customized for the intended recipient). This is, once again, captured in Figure 4 under the category of “navigational assistant.” Sketched maps are much more common as a mode for delivering instructions in the Asynchronous type than the Synchronous type. As we explained above, however, we do not discuss the provision of wayfinding instructions further in this paper, whether Synchronously or Asynchronously delivered. Thus, we have little more to add here to our analysis of Strong Asynchronous social wayfinding. We do observe that both Synchronous and Asynchronous Strong social wayfinding involving instructions supplied by a non-traveler—an “outsider”—are clearly less “democratic” forms of aided wayfinding than Strong wayfinding usually is. Supplying instructions to others typically includes one person *with knowledge* assisting a second person (or group) *without knowledge*, but without actually accompanying the second person (or group) during travel.

### Weak Synchronous Social Wayfinding

In contrast to Strong social wayfinding, other people can influence wayfinding decision-making in indirect and unintentional ways, which we call Weak social wayfinding. When this Weak influence expresses itself during the actual travel of a navigating person, it is not only Weak but also Synchronous. This type is reminiscent of the observation by Munro (cited in Munro et al., 1999): “*[W]e find our ways through spaces from talking to or following the trails of crowds of people*” (Preface, pages unnumbered).

Consider a couple of questions. Have you ever stepped off a train or emerged at an airport gate and blithely followed the dominant flow of people, only to discover that they were leading you somewhere other than to your intended destination? Have you ever hesitated to walk down a street because of the presence of a group of people that seemed somehow, unexpectedly, “out of place” and hence potentially threatening? These situations suggest to us to a basic hypothesis, originally formulated by Dalton et al. (2011): people in un-crowded, relatively sparse environments (i.e., the “normal” milieu) influence where others go, and they do it in one of two ways that we term “person-place cues” and “person-space cues.”

The distinction between person-place cues and person-space cues is subtle but we think it is worth making. The notion of “person-place cues” holds that the location of people in an environment suggests the popularity of that specific place. More people will visit a place that more people want to visit, in other words. This might be due to a temporary event occurring at that place, for example, a street performance artist, or more permanent activities located there, such as shops. People consciously or unconsciously “read” this inferred popularity and make travel decisions accordingly. This inference is more likely to affect route decisions during exploratory-type behaviors such as when one is drifting through an unfamiliar city, wandering around an art gallery, or making spontaneous shopping trips. It is used to judge restaurants: if you are a stranger to a city and are trying to decide which of two restaurants to eat in, do you pick the relatively full one or the empty one? It is clear from this example that we often equate the presence of other people as indicating the popularity and, by extension, even the quality of a place. Hence the term person-place cues.

In contrast, as “person-space cues,” more people in an area imply locations in a spatial layout (a city, a public building) that are better connected to the rest of the layout and more centrally located. Not that the specific place is more popular, but that the rest of the layout is spatially more accessible from that place and, hence, will tend to contain more people over time. The idea of people-space cues is inspired by space syntax theory (Hillier and Hanson, 1984; Bafna, 2003). Space syntax suggests that people pick up visual cues leading to accurate inferences about a space’s importance within the surrounding environment. So-called “integrated” streets readily connect to many other streets and locations; “segregated” streets do not readily lead to many other streets or locations. We read these cues largely unconsciously, having gradually learned their significance through our cumulative experience in similar environments. There will generally be a linear correspondence between the numbers of people walking along a street and that street’s relative status (degree of integration) within the overall settlement (village, town, or city). However, a mismatch between the perceived spatial hierarchy of a street and the number of people present (the pattern of occupancy) will be read as being a somewhat strange or unexpected phenomenon.

We note the causally reciprocal nature of the interrelations between the presence of people, the popularity of places, and the integration/segregation values of streets. For instance, over time, more integrated locations will attract shops, which, in turn, will attract more people; thus, a multiplier effect takes place. This presents a considerable challenge to developing an empirical framework for testing the effects of person-place and person-space cues in naturally existing built environments.

Finally, many of the phenomena of Weak Synchronous social wayfinding in humans relate to a research tradition on “collective navigation” within the study of animal behavior. Larkin and Walton (1969) discussed the idea that fish swimming as members of a school will orient more successfully than the individual fish, as long as the individuals have some orientation ability and obey a simple heuristic to follow the groups’ mean direction. Furthermore, as a result of statistical properties of the errors of individual direction estimates compared to the group mean estimate, this collective advantage will increase with increasing school size. The phenomenon harkens back to Galton’s demonstration in 1907 (Galton, 1907) that there is “wisdom in crowds”; he demonstrated this by showing that the mean estimate of the weight of an ox by a group of individuals was more accurate than almost all of the individual estimates. The superiority of average group decisions over individual decisions based only on simple rules of group aggregation or cohesion is also known as the “many wrongs principle” (Simons, 2004; Berdahl et al., 2016). Faria et al. (2009) directly examined the many wrongs principle with humans, in the context of orientation decisions. They had individuals and groups of 2–10 people attempt to walk toward hidden targets in a circular arena 5 m in radius. Given the simple instruction to stay together, the researchers found improved group performance (lower angular error) over individual estimates, but only for certain group sizes and initial degrees of individual uncertainty. It would clearly be fruitful to further investigate the many wrongs principle for group wayfinding decisions by humans. Undoubtedly, many phenomena of Weak Synchronous social wayfinding are instances of collective navigation as non-human animal researchers conceive of it. However, we note that none of those who research non-humans assign complex cognition to their research subjects. We recognize that humans often act on the basis of simple heuristics, habits, and other unconscious processes, but we also know that humans sometimes act on the basis of complex internal representations, explicit reasoning processes, and the like.

### Weak Asynchronous Social Wayfinding

As is true for Strong social wayfinding, Weak social wayfinding can be manifested asynchronously as well as synchronously. How can people serve as wayfinding cues without doing so during the time a navigator moves through the environment? The answer is that the presence of other people often leaves telltale physical traces of their presence, movement, or other actions within the environment. These traces are available for navigators to read after—sometimes long after—the people who made the traces have left the scene.

**FIGURE 3:**
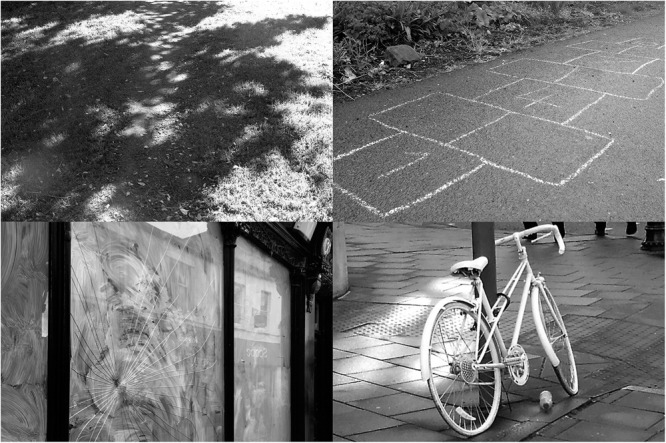
Examples of physical traces of human activity in the environment that could inform the wayfinding decisions of navigators (bottom right photograph of a ‘ghost bike’ courtesy of Damian Cugley, 2007).

Despite *not* being a paper on the topic of wayfinding, the variety and richness of potential physical traces is well summarized by Zeisel (1981), who describes four broad categories: By-Products of Use, Adaptations for Use, Displays of Self, and Public Messages. These are listed in Table 2, with specific sub-types of each broad category indicated in parentheses. We do not review these in detail here (see instead, Zeisel, 1981), but it will serve our discussion to go over some examples that are especially relevant to social wayfinding. The most obvious example is By-Products of Use we refer to as “social trails.” Social trails (or “desire lines”) are the visible paths, often across grass (easily abraded), created by the repeated footfall of past pedestrians taking “shortcuts” through the environment. The presence of such a trail tells us something about the spatial structure of our immediate surroundings and of the activity patterns of other people. It says such a shortcut exists and that by following the path, we might discover an efficient route to a place to which other people have previously traveled, and to which we may want to go. Social trails are clearly the Asynchronous version of Weak social wayfinding, specifically people-space cues. Such social wayfinding is indirect, distal, non-verbal, and created by strangers in sizable numbers. See the upper left quadrant of Figure 3 for an example of social trails.

**Table 2:** Types of physical traces in the environment that wayfinders can use to infer the locations and nature of prior activities by other people.

Types of traces
By-Products of Use (erosions, leftovers, missing traces)
Adaptations for Use (props, separations, connections)
Displays of Self (personalization, identification, group membership)
Public Messages (official, unofficial, illegitimate)

*Adapted from Zeisel (1981).*

We cite and discuss Zeisel’s work here only to provide rich examples of physical traces of human activity in the environment that could inform the wayfinding decisions of navigators. Zeisel’s book itself was not specifically concerned with navigation or social cues. Those listed in Table 2 include traces of occupation or events other than locomotion. Some of these are the asynchronous version of people-place cues, i.e., they say something about the attractiveness of a place rather than the spatial structure of a movement network. Examples might include evidence of children playing (hopscotch drawn in chalk on the sidewalk/pavement), leading us to conclude that this route is part of a residential neighborhood and is probably fairly safe. Broken shop-windows might give the opposite impression of danger: a wayfinder might naturally avoid routes with broken windows and graffiti, deeming them potentially unsafe. In the bottom right section of Figure 3, we see an image of a “ghost-bike,” which signals that a cyclist has been involved in a fatal accident at this location. Although the sighting of a ghost bike might not influence travelers to avoid a route, it might make them more conscious of the traffic dangers and induce them to cross the street using an official crosswalk/pedestrian crossing. These examples make clear that for someone finding his or her way, it is not only the direct presence or absence of people, or explicit navigational instructions, that contribute to route-choice decisions; so can traces of prior movement/occupation.

### Weak and Strong Social Wayfinding Combined

Throughout this paper we have dealt with Weak and Strong social wayfinding separately for sake of clarity. However, it should be evident that it is possible for both types to take place simultaneously. For example a couple might be navigating through a strange city, mutually making wayfinding decisions when required (Strong Synchronous) whilst also attempting to follow an incomplete sketch-map drawn for them previously by a third-party (Strong Asynchronous). Suddenly they notice a large group of people emerging from a building on the other side of an urban square and surmise that this is most likely the transit station they are seeking (Weak Synchronous). Rather than walk around the perimeter of the square, they then notice a worn path that leads directly across the middle of the square and decide to follow this social trail (Weak Asynchronous). This scenario illustrates a range of different ways in which other people contribute, directly or indirectly, to wayfinding decision-making processes and outcomes.

## Integrating Social Wayfinding Into Existing Wayfinding Taxonomies

Finally, we consider how to integrate social aspects of wayfinding into existing models of wayfinding. A number of classifications of navigation behavior have been proposed in the literature (e.g., Kuipers, 1978, 2000; Allen, 1999; Mallot, 1999; Montello, 2001, 2005) many of which were reviewed methodically in a paper by Wiener et al. (2009). In this paper, Wiener et al. use Montello’s definition of wayfinding (Montello, 2001, 2005) as a starting point to develop a taxonomy of wayfinding that concentrates on how different levels and degrees of spatial knowledge (about landmarks, routes, survey relationships) fundamentally structure the nature of a wayfinding task and the likely solution strategies that will be applied to carry it out. In this model (as in all others) the wayfinder is treated as an individual and no mention of social factors is given; this is, therefore, the taxonomy of wayfinding that we selected in order in order to determine whether or not we could augment it with additional features of social wayfinding.

**FIGURE 4:**
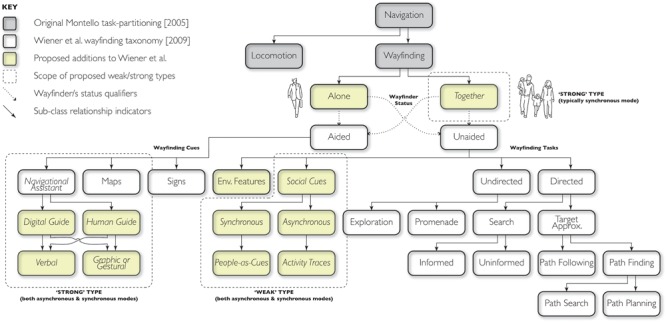
Proposed additions to Wiener et al. (2009) wayfinding taxonomy indicating where the strong/weak and synchronous/asynchronous types of social wayfinding would fit.

Based on the typology of social wayfinding we provide in this paper, the Wiener et al. (2009) taxonomy can be extended (see Figure 4 showing how our framework might augment their original taxonomy) at two levels: just as wayfinding can be separated into aided and unaided, the wayfinder can be alone or can travel as part of a group of collaborative wayfinders. The standard case of “together” wayfinding would be a group of two or more people who jointly aim to find their way from A to B, without using explicit navigation aids. Each individual can be characterized in terms of his or her degree of active “wayfinding involvement” in the decision-making process, to any extent from fully to not at all. Full involvement would be involvement in the wayfinding decision-making to the same extent as if he or she were wayfinding alone, completely responsible for the decision. No involvement would be equivalent to quietly acquiescing to whatever others decide (tourists following a guide or children following their parents). Quantifying degrees of involvement would be an interesting and challenging research task, but it seems conceptually coherent to see it as a matter of the number and nature of comments the individual makes to the group, the passion or confidence brought to the discussion, and so on.

Related to the issue of each individual’s involvement, any wayfinding group can be characterized in terms of the degree to which responsibility for wayfinding decisions is shared equally among group members or concentrated in an incomplete subset of the group, often a single person. We propose to refer to this as the “wayfinding egalitarianism” of the group, ranging from autocratic to fully egalitarian. In the autocratic group, one individual makes the wayfinding decisions for the group entirely on his or her own. In the perfectly egalitarian group, each individual contributes equally to the wayfinding decisions of the group. By using these terms, we do not mean to imply that the distribution of wayfinding responsibility within a group is necessarily a matter of fairness or social justice. In many cases, people are happy, even grateful, to let someone take responsibility for wayfinding decisions, or only a subset of group members have information relevant to wayfinding decisions. However, there will certainly be situations where more confident or domineering group members assume control over wayfinding decisions, without assent from other group members.

There are two types of social wayfinding we have discussed that can readily be identified as part of the Aided Wayfinding side of the Wiener et al. (2009) taxonomy. We also classify these as part of Strong social wayfinding. These are when the social other explicitly provides wayfinding instructions to guide the travelers without wishing to travel to the destination with those travelers (“human guide” in Figure 4). As we have seen, wayfinding instructions, sketch maps, and gestures can be supplied synchronously or asynchronously. Of course, Aided Wayfinding in the Wiener et al. taxonomy also includes commercially produced maps and signs. As we discussed above, we recognize the sociocultural nature of these semiotic artifacts, but have not discussed them in this paper because they are not tailored to specific people or specific trips. By contrast, we do treat the “navigational assistants” that Wiener et al. include in this category as cases of social wayfinding, not only because people originally created and programmed the systems but because the systems do create wayfinding instructions tailored to specific origin-destination trips.

The relationship of our Weak social wayfinding types to the Wiener et al. taxonomy is interesting and not obvious. We lean toward considering them as Unaided Wayfinding. Wayfinding aids such as maps, signs, and verbal instructions are intentionally created “semiotic artifacts” (Montello and Sas, 2006) designed to provide wayfinding information and guidance. The presence of others (Weak Synchronous) and physical traces of their past presence (Weak Asynchronous) are not semiotic artifacts—they are people who are, or were, doing their own thing. They are analogous to traditional landmarks, environmental features that can (and very often do) provide cues to orientation without having been intentionally created for that purpose. In contrast, a sign *acting as a sign*, rather than a memorable object, is not a landmark but a semiotic artifact. We do want to highlight the special nature of other people as environmental cues, both people-place and people-space, so we propose to add a category to the taxonomy of Unaided Wayfinding we call “Social Cues.”

Finally, Wiener et al. (2009) explicitly point out that their taxonomy does not imply a real-world wayfinding task should always be classified into only one of the categories in the taxonomy. For example, a traveler may start out in exploration mode when window-shopping in the city, but once she or he wants to get back to the car, the trip turns into path search or path planning (depending on how much attention was paid to memorizing the travel during exploration).

## Conclusion

Other people can act in three broad roles as part of wayfinding: as co-decider, route instructor, or environmental cue. There are significant gaps in our understanding of social wayfinding. It is without doubt an under-researched area. It poses a host of interesting but difficult research issues, made all the more complex by the emergent properties of groups of interacting individuals, and by the number of different academic disciplines we believe are required to investigate these phenomena. The role of social factors in wayfinding is important both for basic research and for applications. An example of application is the growing area of developing and evaluating digital navigational aids (such as mobile/handheld navigational assistants). The importance of social wayfinding is clearly relevant to the user experience and functionality of these aids. The growing research on indoor navigation (e.g., Frankenstein et al., 2012), such as shopping centers and airports, is another area where social ideas area particularly relevant.

Our understanding of the mechanisms and information sources contributing to decision-making during wayfinding needs to include social aspects to become comprehensive and ultimately deliver a complete model of wayfinding behavior. On the applied side, this understanding can inform architectural planning, signage design, and the development of digital devices such as mobile maps, both by translating theoretical and empirical findings into design decisions, and by providing an enriched simulation model for capacity planning, building evaluation, etc. Crowd-level pedestrian dynamics research (see Helbing et al., 2000; Moussaid et al., 2009) as well as development of commercially available simulation models has been focused on lower-level perceptual and motoric phenomena. Currently such systems are very successful at predicting navigation at the level of locomotion rather than wayfinding. This includes avoiding collisions, bypassing other slower people, forming lanes and crowd-level congestion dynamics. Simulating wayfinding qualities of buildings, neighborhoods, or signage/map systems would require taking into account cognitive factors such as the goals of the navigators, group membership, social relations, and spatial mental representations underlying wayfinding decision-making.

We are led to conclude that contrary to the assumption (whether implicit or explicit) underpinning the great majority of the existing literature on wayfinding psychology (including Wiener et al.’s taxonomy), wayfinding in many—perhaps most—real-world cases has a critical social dimension to it. “Asocial wayfinding” happens mostly in artificially controlled situations, such as in psychology experiments or computational models. When one considers the widespread adoption of navigation assistants currently underway, at least in the developed world, it is clear that navigation uninfluenced by others is becoming even less common. Paradoxically, this leads people to ask other people in the surrounds for directions less and less, and to ignore people-as-cues more and more.

## Author Contributions

RD originally conceived of the main conceptual ideas and further developed the theoretical framework in discussions with CH. Later RD and CH joined with DM who had been working on similar ideas in parallel, but independently. In general, this work builds upon, and extends, previous work by all authors. All authors contributed to the final version of the manuscript.

## Conflict of Interest Statement

The authors declare that the research was conducted in the absence of any commercial or financial relationships that could be construed as a potential conflict of interest.

## Footnotes

1 We recognize that some authors have used the term “navigation” as synonymous with our use of “wayfinding” (e.g., McNamara et al., 2008; Ekstrom et al., 2018).

2 This research area is often called “social navigation” in the HCI community. The navigation is metaphorical, however, as it refers to travel through “information” space. It is an ongoing research issue as to how closely processes of navigation in literal and information spaces correspond. We expect that some of the processes observed in one type of space will also be observable in the other; we also expect they differ psychologically in important ways.

3 Of course, when the sender is a computer navigation system, we can consider the information exchange intentional but probably want to avoid saying the system is “aware” of what it is doing.

4 We do not address familiarity *explicitly* in this paper, however, it is *implicitly* built-in, as it forms a key component of Wiener et al.’s taxonomy, which we expand, at the end of this paper (2009). In their original model they assume, as do we by extension, that familiarity with an environment certainly influences wayfinding strategies.

5 Although preliminary findings for the crowd-sourced, navigational-data game, Sea Hero Quest, indicate that wayfinding performance is 11% better in men than women across all age groups (Spiers et al., 2017).
